# Transcriptional Analysis Reveals Different Fertilizer Treatments Improve the Yield and Quality of Tomato Fruits by Postponing Age-Triggered Senescence

**DOI:** 10.3390/plants15192990

**Published:** 2026-09-30

**Authors:** Xiaodong Yang, Shasha Sun, Lingling Huang, Jun Xu, Kun Xie, Bo Zhou, Fengjuan Yang, Jing Li

**Affiliations:** 1 Weifang Academy of Agricultural Sciences, Weifang 261071, China; 2 College of Horticulture Science and Engineering, Shandong Agricultural University, Tai’an 271018, China; 3 College of Life Science, Shandong Agricultural University, Tai’an 271018, China; 4 Shandong Key Laboratory of Fruit and Vegetable Germplasm Innovation and Utilization, College of Horticulture Science and Engineering, Shandong Agricultural University, Tai’an 271018, China; 5 Key Laboratory of Biology and Genetic Improvement of Horticultural Crop (Huang-Huai Region), Ministry of Agriculture and Rural Affairs, Tai’an 271018, China

**Keywords:** tomato, quality, yield, fertilization, age-triggered senescence

## Abstract

Inappropriate fertilization has led to adverse environmental effects and food-safety issues. Biostimulants have been widely used to improve the growth, yield, and quality of crops. However, the effects of biostimulants on age-triggered senescence in plants, which affect the later yield and quality, have scarcely been reported. In this study, the fertilization formulas that were modified according to the requirements of plants in different growth stages, and adding yeast extract agents or microorganisms were respectively used in soilless tomato culture as three fertilization treatments. The Yamazaki formula was set as the control. As results, leaf senescence was delayed by the three fertilization treatments, and the yield and quality of the tomatoes were improved. RNA-seq analysis was used to identify the underlying regulatory mechanisms. The results showed that the genes expression levels remained unchanged in leaves in response to the three fertilization treatments. However, the “MAPK signaling pathway—plant” and “Plant-hormone signal transduction” processes between the roots and leaves were significantly affected, including auxin, gibberellin, ethylene, jasmonic acid, cytokinin, brassinosteroid, and the salicylic acid regulatory pathways. In addition, senescence-related transcription factors were significantly differentially expressed between roots and leaves, including WRKY, MYB, and AP2-EREBP. Our results confirm that using three fertilization formulas can improve the tomato yield and quality, and indicate that a close association exists between root and leaf senescence.

## 1. Introduction

The green revolution has resulted in enormous agricultural productivity, which is mainly achieved with the use of synthetic agrochemicals and fertilizers [1]. However, the excessive and continuous use of synthetic agrochemicals and fertilizers has led to detrimental side effects on human health and food safety, including food contamination, negative effects on the environment, and disease resistance [2,3,4]. The challenge of global food security is straightforward: by 2050, the world’s population will reach 9 billion people, and the demand for food will increase by 60%. Thus, it is very important to develop new approaches to produce sufficient quantities and quality vegetables in a protracted socio-economic crisis. Moderate use of agrochemicals and fertilizers or using biostimulants are two effective approaches to sustainably improve farm productivity and vegetable quality. 

Fertilizer applications provide various nutrients for growth, and maintain or improve soil reserves [5]. However, many factors affect the requirements of vegetables for fertilizers, including the soil type and nutrient content, the length of the growing season, and the growth stage [5]. Many nutrient manufacturers have developed special formulas optimized for different growth stages of specific plants, such as tomato (*Solanum lycopersicum* L.). These formulas not only improve vegetable quality but also reduce the amount of fertilizer used [6].

Microorganisms, an important group of plant biostimulants, have been shown to increase vegetable growth [7], nutrient uptake, and yield [8] via different mechanisms, such as by increasing nutrient solubility and the symbiotic relationships in the soil [9]. Soil microorganisms play fundamental roles in agriculture, and are an important factor determining plant growth and vegetable productivity worldwide [1,10,11,12]. Previous studies have reported that microorganisms and plants communicate through molecular signals, and most of the plant–microbe communication occurs in the rhizosphere within a millimeter of the root surface [1,10,11,12]. This intercommunication dictates the growth and development of plants [2], and a reciprocal relationship has developed between plants and associated microorganisms. However, this reciprocal relationship is inadvertently broken during plant breeding, resulting in the loss of key beneficial members of the plant microbiome [13]. Therefore, establishing specific plant microbiomes using effective microorganisms is an environmentally friendly technology to ensure vegetable safety and quality. 

In the last decade, many studies have reported that microorganisms can be used as an amendment to enhance soil fertility when cultivating crops, increase crop yield [14,15,16], and defend against abiotic stress [2,17,18,19,20,21]. However, most of these studies focused on the change in physiological indicators in plants and the rhizomicrobiome environment in the soil after using microorganisms [2,9,14,17]. Few studies have systematically explored the potential changes in molecular signals in the aboveground and underground parts of plants in response to microbes [22], particularly in the context of delaying plant senescence.

Tomatoes are a rich source of minerals, vitamins, and antioxidants, and demand a leading global cultivation area worldwide [23]. Liquid fertilizer and the Yamazaki formula are commonly used in soilless tomato culture. However, Kinoshita et al. [24] reported that using a controlled-release fertilizer application system can reduce nutrient use by 37%, and the fruit yield is comparable to that cultivated with an open liquid fertilizer system. Our preliminary work also demonstrated that moderate reductions in total nitrogen (N), phosphorus (P), and potassium (K) during soilless tomato culture improve tomato yield and quality. In addition, Ndona et al. [25] reported that microorganisms combined with a stone dust suspension increase the percentage of best-quality tomato fruits. Mahjoob et al. [26] reported that an application of effective microorganisms increases tomato marketable yield by 11.8–30.7% over a control. 

In this study, a fertilization formula that was modified according to different growth stage requirements of tomatoes, and the addition of yeast-extract agents or microorganisms to the Yamazaki formula delayed plant senescence and improved the yield and quality of the tomatoes. Moreover, the underlying regulatory mechanism was explored by RNA-seq analysis. The results revealed that the signal transduction processes between roots and leaves of tomatoes were significantly affected. Our study provides a theoretical basis for postponing age-triggered senescence in tomato plants.

## 2. Results

### 2.1. Major Agronomic Traits and Biochemical Analysis of Tomato Plants Under the Different Treatments

**Figure 1:**
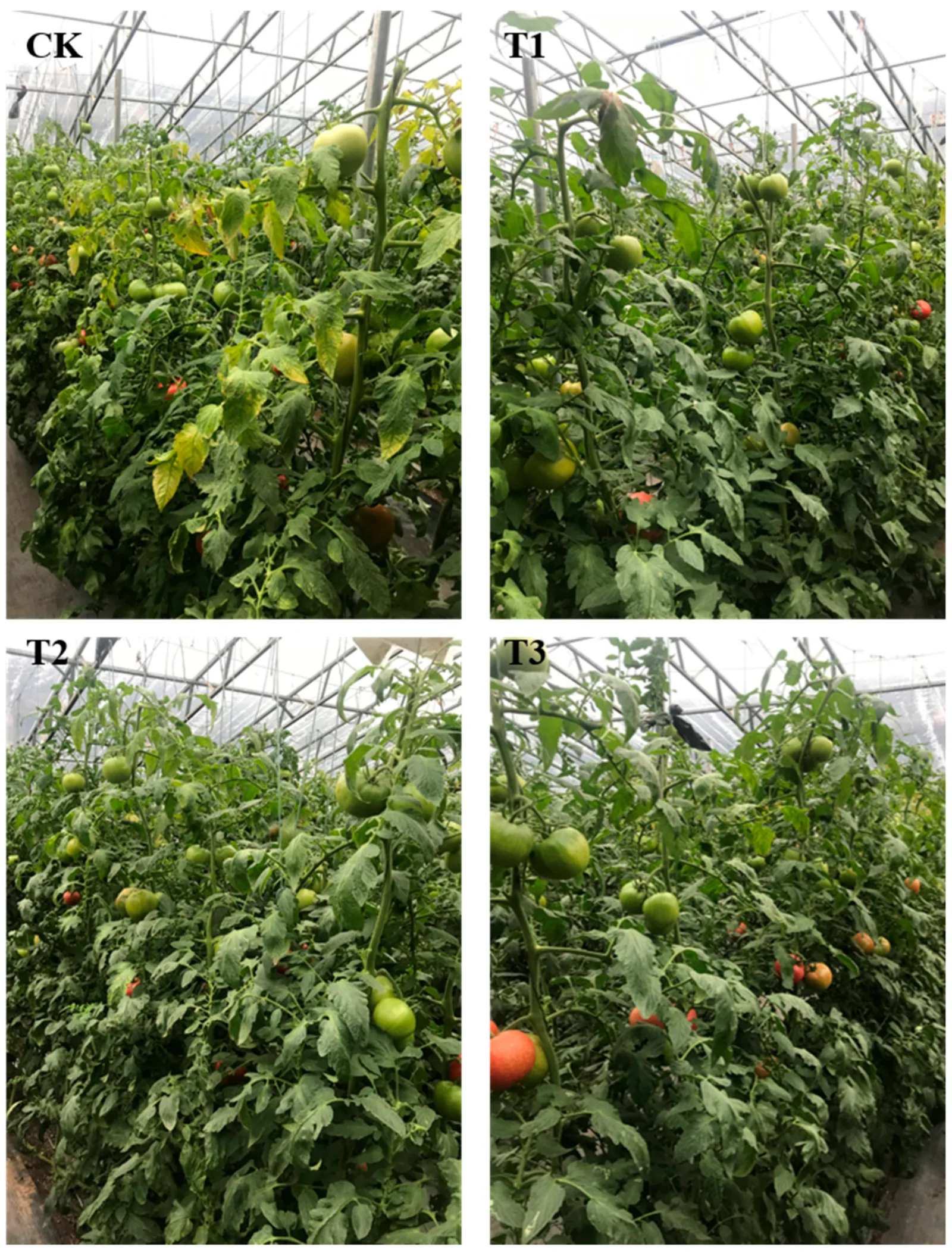
Plant phenotypes of tomato under different fertilization treatments at the plant-topping stage.

**Figure 2:**
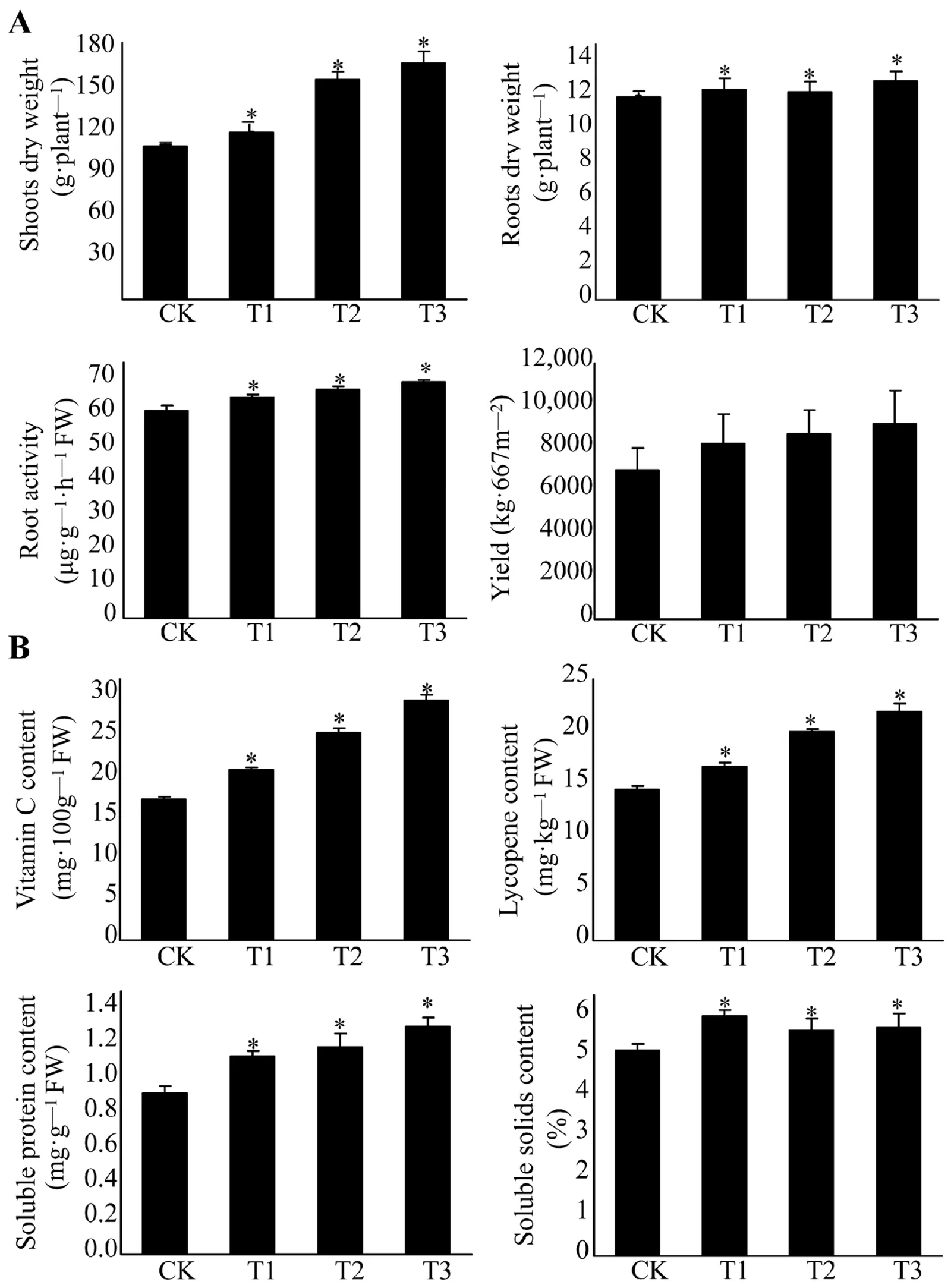
The physiological indicator value of tomato under different fertilization treatments. (**A**) The shoot dry weights, root dry weights, root activity, and yield of tomato; (**B**) The contents of vitamin C, Lycopene, soluble protein, and soluble solids in the completely red and ripe tomato fruits. *: indicates significance compared to the control at the 5% level, and bars represent the standard errors.

As shown in Figure 1, the leaves under the control (CK) treatment displayed obvious yellowing, while the leaves under the treatments (T1, T2, and T3) remained green. Then, the aboveground and underground dry weights, root activity, yield, and quality parameters, including the contents of soluble solids, vitamin C, soluble protein, and lycopene in tomato fruits were measured. These parameters differed between the CK and the three treatments. Except for soluble solids, the other parameters were the highest under the T3 treatment, followed by T2, T1, and CK (Figure 2). These results suggested that the yield and quality of the tomatoes were increased by three fertilization treatments compared with CK, particularly by the T3 treatment.

### 2.2. RNA-Seq Analysis and Identification of Differentially Expressed Genes

**Figure 3:**
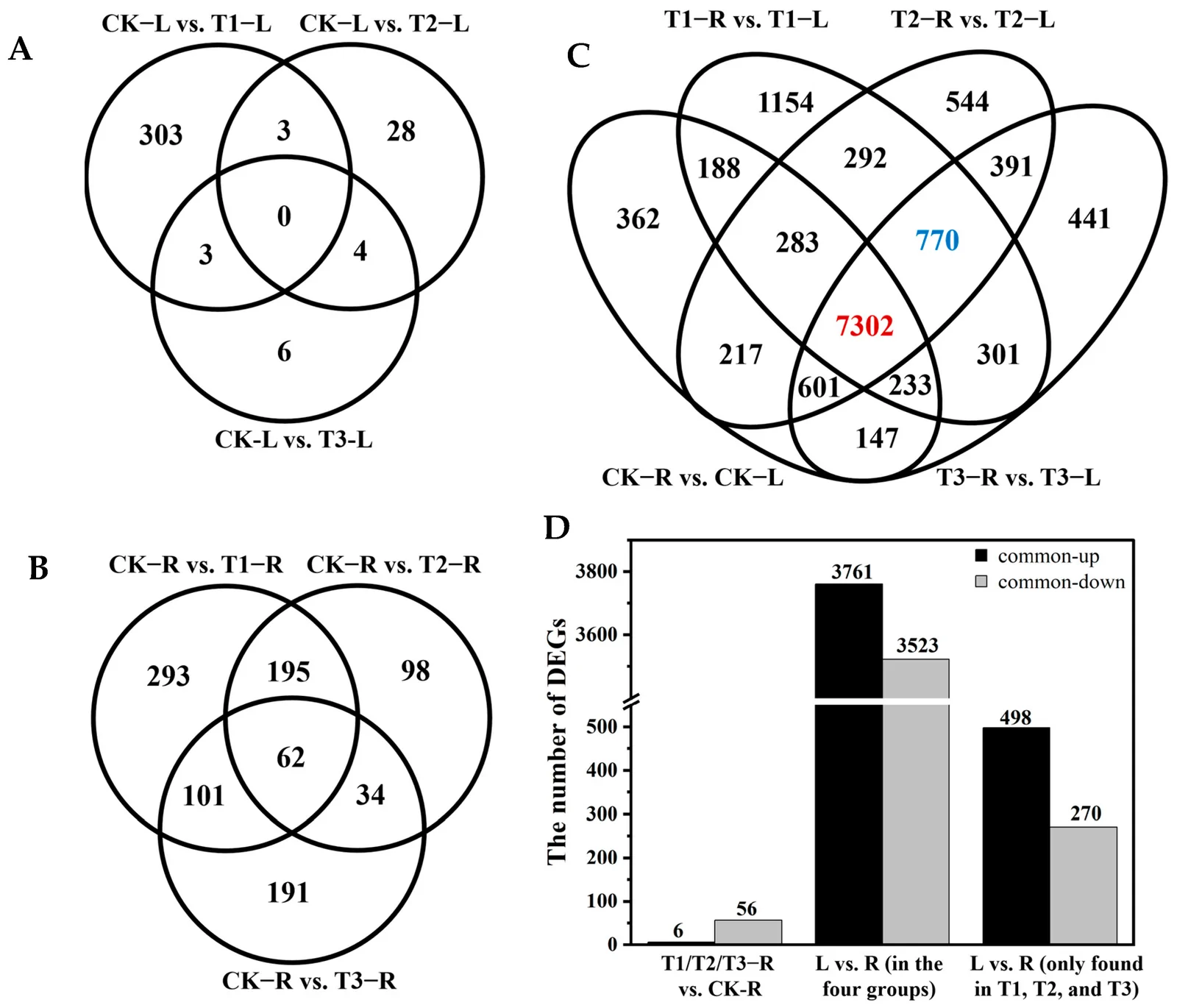
Venn diagram analysis of common DEGs in the leaves and roots under different fertilization treatments. (**A**) The common DEGs in the leaves among the comparison group of CK and three fertilization treatments; (**B**) The common DEGs in the roots among the comparison group of CK and three fertilization treatments; (**C**) The common DEGs among the comparison group of roots and leaves under different fertilization treatments; (**D**) The number of DEGs with same expression trend.

To explore the mechanism underlying the postponing age-triggered senescence by the different fertilization treatments, RNA-seq analysis was conducted on the uppermost node leaves (L) and roots (R) from the CK, T1, T2, and T3 treatments. Twenty-four sample libraries were sequenced in total (Table S1). After removing the sequencing adapters and low-quality data, the clean reads ranged from 20.16 to 23.35 Mb in the different treatments, and the Q20 and Q30 percentages of all treatments were >97% and 89% respectively, demonstrating the high quality of the sequencing data. Totals of 78.66–88.06% and 68.66–79.2% of the total reads from the RNA-seq data were uniquely mapped to the genome and genes, respectively (Table S1). A Q-value (adjusted *p*-value) ≤ 0.05 and |log_2_ (fold change)| ≥ 1 were used as the threshold criteria to identify the differentially expressed genes (DEGs). Notably, only 309, 35, and 13 DEGs were found in the leaves under the T1, T2, and T3 treatments, respectively, compared with CK-L, and no common DEGs were found (Figure 3A). However, compared with CK-R, 651, 389, and 388 DEGs were found in the roots under the T1, T2, and T3 treatments, respectively, and 62 common DEGs were found (Figure 3B). Among the 62 common DEGs, 56 genes were downregulated (named as 56 common downregulated DEGs) while six genes were upregulated (named as six common upregulated DEGs) in the roots under the three treatments compared with CK (Figure 3D). Considering that the treatment conditions were only different in the tomato roots, but the aboveground parts also showed different performance, we guess that the roots could regulate gene expression in the aboveground tissues through signal transduction. Therefore, the changes of the DEGs between the roots and leaves were analyzed. As shown in Figure 3C, 7302 genes were differentially expressed between the roots and leaves of the CK and the three treatments, and only 18 genes had different expression patterns among the four groups (Figure 3D). In addition, 770 DEGs were only found in the T1-R vs. T1-L, T2-R vs. T2-L, and T3-R vs. T3-L. Among the 770 DEGs, the expression levels of 270 genes were significantly lower in leaves than those in roots (named as 270 higher expressed genes in roots), while the expression levels of the 498 genes in leaves were significantly higher than those in roots (named as 498 higher expressed genes in leaves) under the three treatments (Figure 3D).

### 2.3. Functional Classification of the DEGs by Enrichment Analysis

Gene Ontology (GO) enrichment analysis was carried out to identify the major functional categories of the above DEGs. As shown in Figure S1, the four kinds of DEGs were all classified into biological process (GO-BP), cellular component (GO-CC), and molecular function (GO-MF) categories. Moreover, the largest class among the three categories was almost the same among the four kinds of DEGs. In the GO-BP category, the largest class was cellular or metabolic processes. In the GO-CC category, the cellular anatomical entity was significantly enriched in the four kinds of DEGs and was the largest class. Binding and catalytic activity were the two largest classes in the GO-MF category.

**Figure 4:**
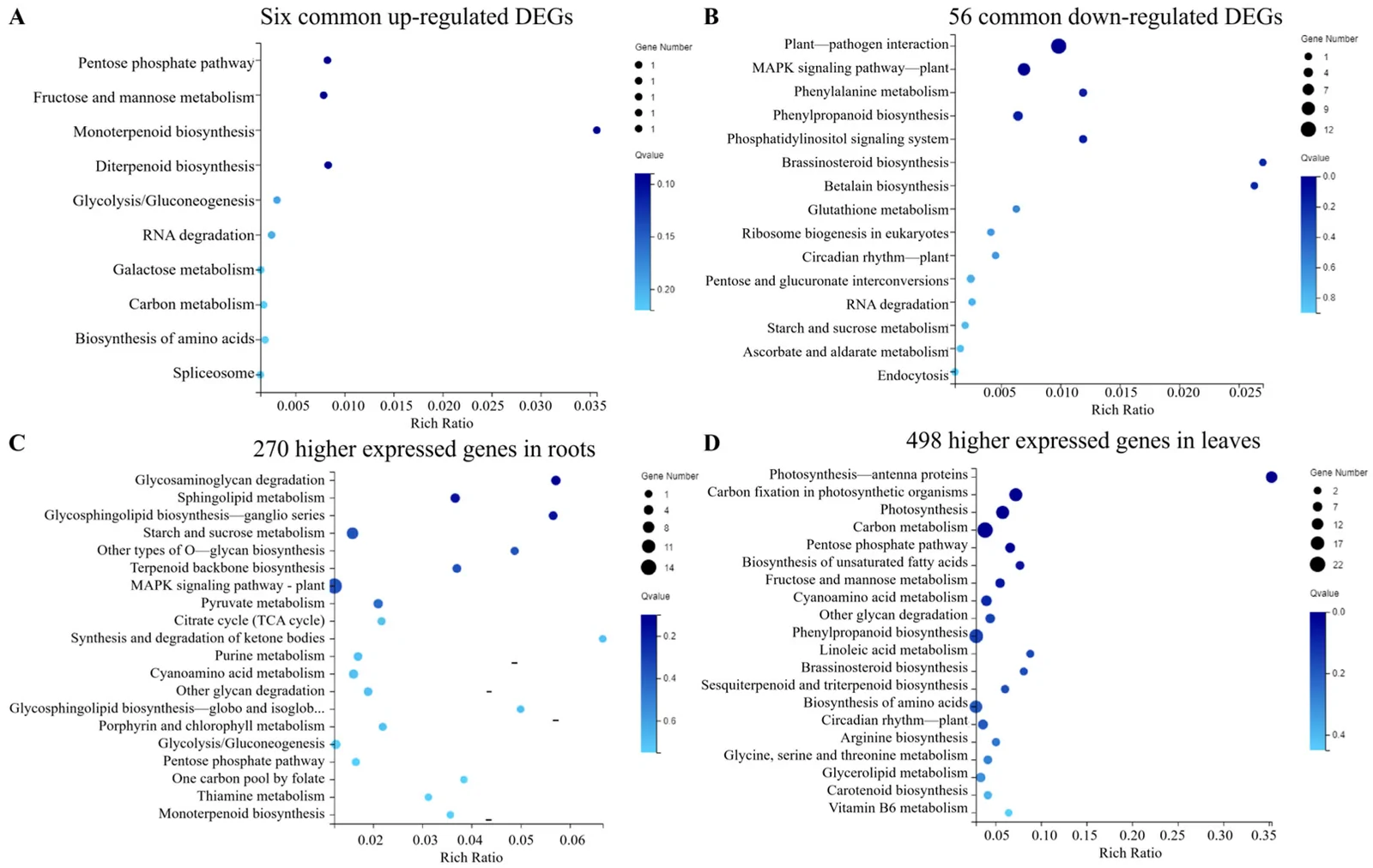
KEGG pathway enrichment analysis of common DEGs with same expression trend. (**A**) KEGG pathways enriched by six common up-regulated DEGs in roots among the comparison group of three fertilization treatments and CK. (**B**) KEGG pathways enriched by 56 common down-regulated DEGs in roots among the comparison group of three fertilization treatments and CK. (**C**) KEGG pathways enriched by 270 higher expressed genes in roots under the three fertilization treatments. (**D**) KEGG pathways enriched by 498 higher expressed genes in leaves under the three fertilization treatments.

To investigate the biological pathways important for higher yield and quality of tomatoes under the three treatments, the above DEGs were further annotated based on the reference pathways in the Kyoto Encyclopedia of Genes and Genomes (KEGG) database. The “Metabolism of terpenoids and polyketides” and “Carbohydrate metabolism” pathways were enriched in the six common upregulated DEGs (Figure 4A and Figure S2A). The signal transduction pathways including “Plant–pathogen interaction”, “MAPK signaling pathway—plant”, and “Phosphatidylinositol signaling system” were enriched in the 56 common downregulated DEGs (Figure 4B and Figure S2B). The carbohydrate metabolic and signal transduction pathways, including “MAPK signaling pathway—plant” and “Plant-hormone signal transduction” were the most enriched pathways in the 270 highly expressed genes in roots (Figure 4C and Figure S2C). The most enriched pathways in the 498 higher expressed genes in leaves mainly belonged to the energy and carbohydrate metabolic pathways. Meanwhile, the “MAPK signaling pathway—plant” and “Plant-hormone signal transduction” pathways were also enriched (Figure 4D and Figure S2D). Taken the above together, we speculate that the genes involved in the signal transduction pathways may play an important role in the yield and quality formation of tomatoes promoted by the three fertilization treatments.

### 2.4. The DEGs Involved in Signal Transduction

The above DEGs enriched in the signal transduction pathways were furtherly analyzed (Table 1). A total of eight, nineteen, and thirty-five genes involved in the fifty-six common downregulated DEGs, two hundred and seventy higher expressed genes in roots, and four hundred and ninety-eight higher expressed genes in leaves were enriched in the signal transduction pathways, respectively. Six of the eight genes were enriched in the “MAPK signaling pathway—plant” pathway, while none were found in the “Plant-hormone signal transduction” pathway. Eleven of the nineteen genes were enriched in the “MAPK signaling pathway—plant” pathway, while six of the nineteen genes were found in the “Plant-hormone signal transduction” pathway and involved in the auxin, gibberellin, ethylene, and jasmonic acid regulating pathways. 13 of the 35 genes were enriched in the “MAPK signaling pathway—plant” pathway, while 15 of the 35 genes were found in the “Plant-hormone signal transduction” pathway and involved in auxin, cytokinin (CTK), gibberellin, ethylene, brassinosteroid, jasmonic acid, and salicylic acid regulatory pathways.

**Figure 5:**
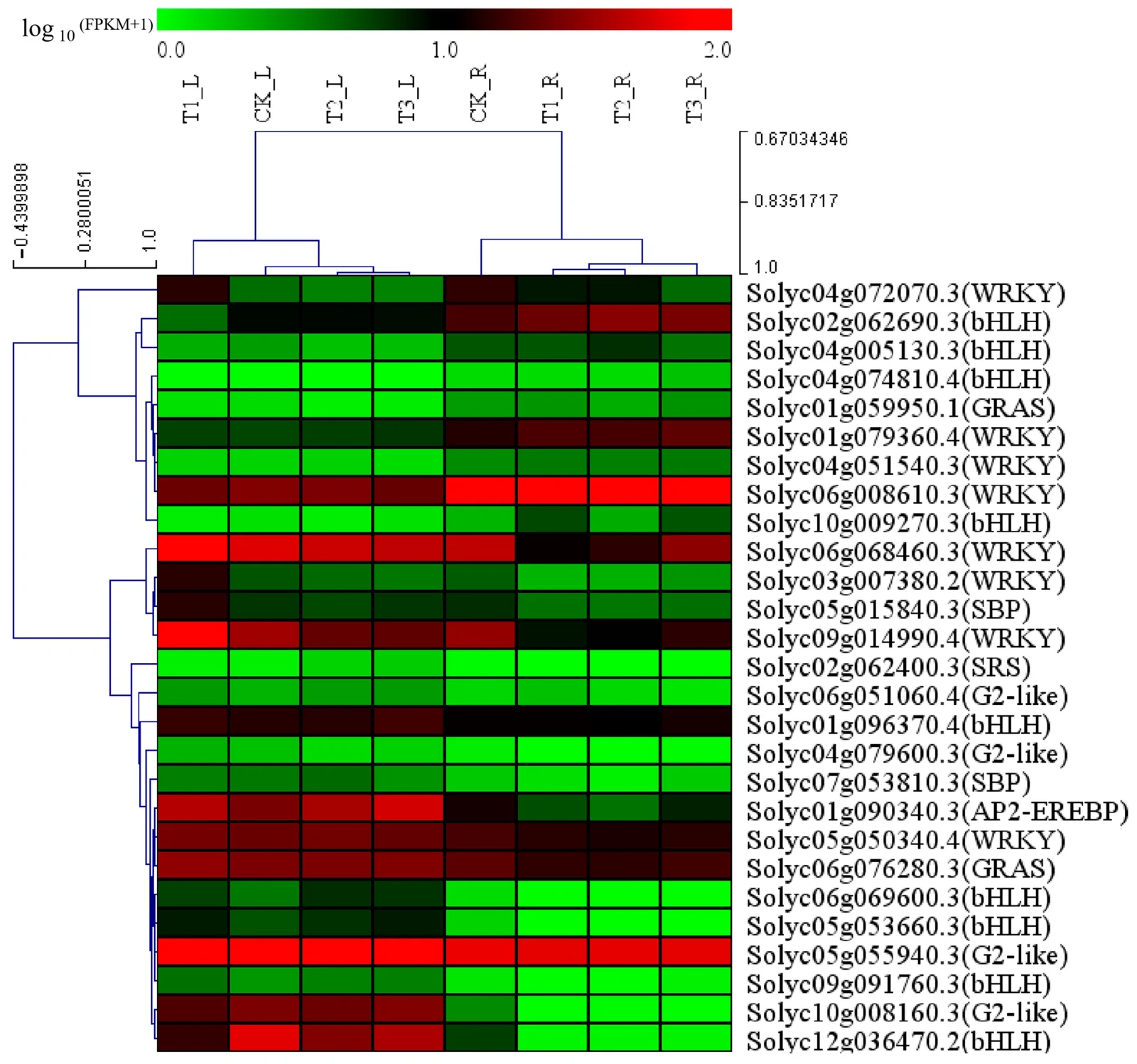
Hierarchical clustering analysis of the transcription factors genes involved in the signal transduction pathway based on the RNA-seq data. Log_10_^(FPKM+1)^ was used for the heat map to normalize the gene expression level. The color scale represents differences in gene transcript levels.

Notably, two, eight, and eighteen genes encoding transcription factors (TFs) were involved in the signal transduction pathways. These TFs included nine bHLHs, eight WRKYs, four G2-likes, two GRASs, two SBPs, one AP2-EREBP, and one SRS. To determine the gene expression patterns of these TFs under the CK and the three treatments, we constructed a hierarchical clustering map (Figure 5). As shown in Figure 5, the four treatments were classified into two classes according to the expression levels of these TFs, and similarities were found among the CK, T2, and T3 treatments. In addition, the expression levels of most TFs in the leaves were significantly different from those in the roots, and only a few TFs showed significantly different expression levels in the leaves or roots among the three treatments. For example, *Solyc04g072070.3* (*WRKY*), *Solyc03g007380.2* (*WRKY*), *Solyc05g015840.3* (*SBP*), and *Solyc09g014990.4* (*WRKY*) were expressed at higher levels in the T1_L and T1_R, while *Solyc02g062690.3* (*bHLH*) was expressed at lower levels in T1_L and T1_R. *Solyc12g036470.2* (*bHLH*) was expressed higher in the CK_L and T1_R, *Solyc10g008160.3* (*G2-like*) was expressed higher in the T1_R, while *Solyc06g069600.3* (*bHLH*) was expressed lower in the CK_L.

### 2.5. Validation of the RNA-Seq Data by qRT-PCR

**Figure 6:**
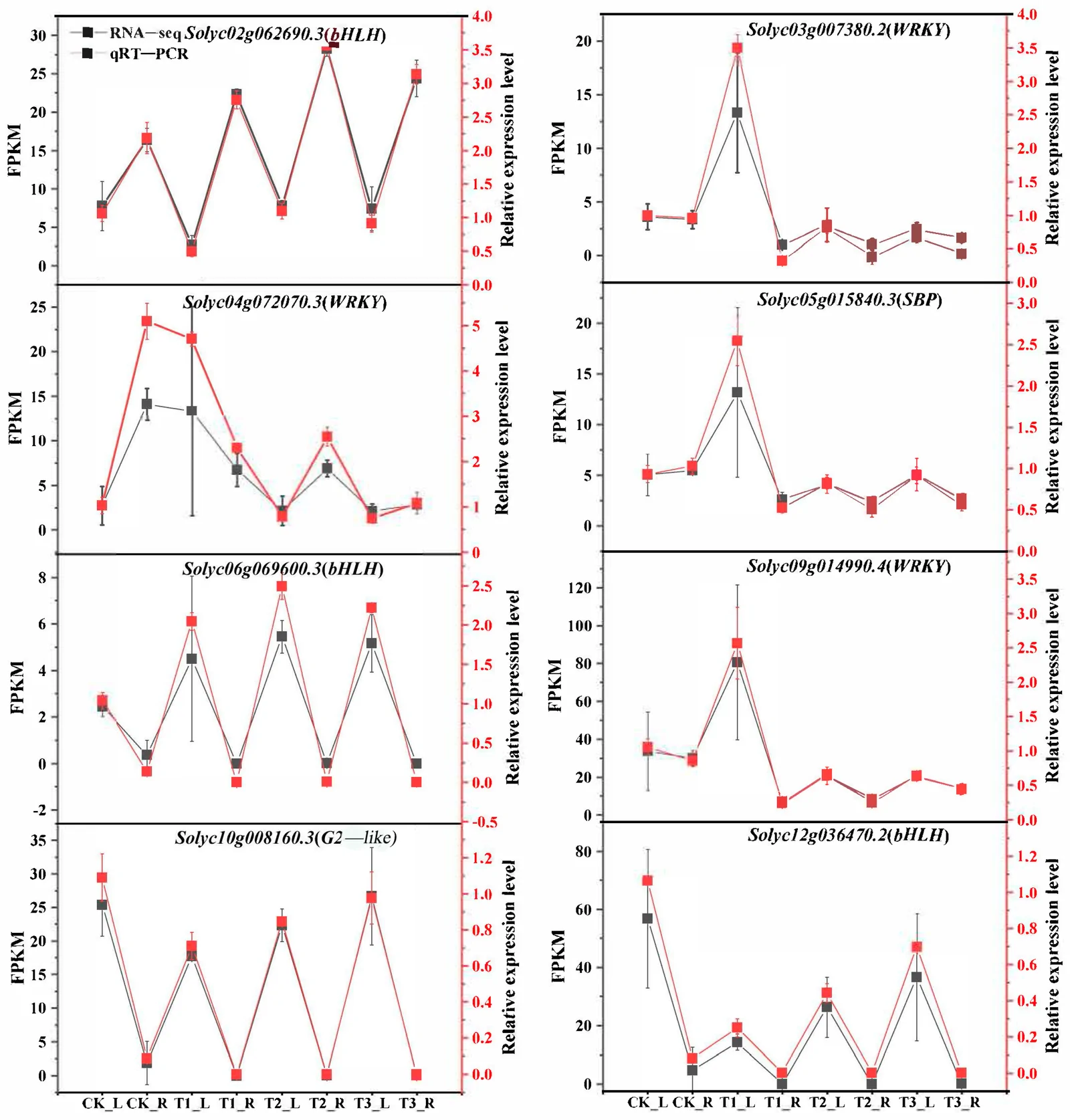
QRT-PCR validation of eight selected transcription factors genes. The data represent the means from three replicates with three biological repeats. Error bars indicate standard error.

In addition, these eight TFs with different expression patterns were chosen to validate the RNA-seq data by qRT-PCR (Figure 6). The qRT-PCR results support the RNA-seq expression patterns of these eight selected genes.

## 3. Discussion

Previous studies have reported that the soil applications of effective microorganisms improve the growth, yield, and quality of crops [9,27]. However, the underlying molecular mechanism is not well known. In this study, we found that the leaves yellowing was obvious under the CK, which was one of the most typically visible signs of plant senescence. However, the leaves under the T1, T2, and T3 treatments remained green at the same stage, suggesting that the modified fertilization formula, and the addition of yeast extract and microorganisms were beneficial to delay senescence in tomato plants. Moreover, the yield and quality of the tomatoes improved. This is similar to the study by Zhang et al. [28], which found that using bag-controlled release fertilizer can promote the growth of fine roots and also delays leaf aging, fruit yield, and fruit quality in peach trees. Premature senescence of plants seriously affects yield and quality [29,30]. Therefore, we speculated that delayed plant senescence might be an important reason for the higher yield and quality of tomatoes under the three fertilization treatments. 

Plant senescence is an age-dependent degradation process and is inevitable. Age-dependent senescence has been widely studied in different plant tissues, particularly in leaves [31]. Although previous studies have described that root browning decreases the nutrient uptake rate and degeneration of individual root cell layers appears over time [32,33,34], research about the senescence process in roots has just begun [35]. Previous studies have found that there are signal transduction pathways between plant roots and leaves, and these signal transductions can influence the growth or stress resistance of plants. Bano’s research indicates that ABA and cytokinins alleviate the damage caused by salt stress to rice through signal transduction in the rhizome [36]. Wang et al. [37] also found that acetylcholine can transmit signals between roots and leaves, thereby regulating the opening and closing of stomata in leaves. Here, we analyzed the gene expression levels in the roots and leaves of tomatoes under three fertilization treatments using RNA-seq. According to the bioinformatics results, few DEGs were found in the leaves under the three fertilization treatments compared with the CK, suggesting that the three fertilization treatments did not directly delay leaf senescence. We speculate that there may be signal transduction between roots and leaves which delays plant senescence and improves yield and quality, particularly in the “Plant-hormone signal transduction” pathway. Compared with CK, the genes involved in the auxin, gibberellin, ethylene, and jasmonic acid regulatory pathways were differentially expressed in roots and leaves and were expressed at higher levels in roots. Simultaneously, the genes involved in the auxin, cytokinin, gibberellin, ethylene, brassinosteroid, jasmonic acid, and salicylic acid regulatory pathways were expressed higher in leaves. Liu et al. [35] assumed that abscisic acid peaks and auxin and CTK levels decrease sharply triggered by the progression of morphological and physiological degeneration in barley root segments. In contrast, Morris et al. [38] reported that salicylic acid plays a role in regulating gene expression accompanied by delayed yellowing during leaf senescence. We discovered that the hormone signals were different during the root and leaf senescence processes, but whether some association exists between them is worthy of further investigation.

The identified DEGs were also significantly enriched in the “MAPK signaling pathway—plant” pathway, which plays pivotal roles in signaling plant defense against pathogen attack [39]. Continuous cropping obstacles affect crop yield and quality by changing the soil microbial community structure, accumulating autotoxins, weakening plant growth, and aggravating diseases and pests [40]. Li et al. [41] reported that the bacterial community, particularly the rhizobacterial community, exhibits a time-decay pattern along with the increase in the cultivation year, while the cultivation year did not affect the fungal microbiome. In this study, substrate cultivation was used for tomatoes, but the quantity, diversity, and richness of the microorganisms in the substrate may have become imbalanced at the later stage of plant growth. Soil microorganisms play important roles in the occurrence and prevention of plant diseases [11,42]. Taken together, we speculate that adding yeast extract agents and microorganisms along with reasonable fertilization improved or recombined the underground microbial community structure. This is a topic that requires further in-depth research. Therefore, the expression levels of genes involved in the “MAPK signaling pathway—plant” pathway were lower in the roots of the three treatment groups compared with the control, suggesting delayed activation of the defense system or that the age-triggered senescence signal released from roots was delayed. 

In addition, the TFs NAC, WRKY, and AP2-EREBP were considered as prominent regulators of leaf senescence [43,44]. In the apical and basal root tissues of barley, several NAC-, WRKY-, and AP2-type TFs were reported to be upregulated just before the arrest of root elongation [35]. In this study, WRKY-, MYB-, MADS-, zf-HD-, C2H2-, and AP2-EREBP-type TFs were significantly downregulated by the three fertilization treatments compared with CK. Moreover, several bHLHs, WRKYs, G2-like, GRASs, SBPs, AP2-EREBP, and SRS-type TFs were found in the signal transduction process, most of which showed the same expression pattern in the leaves and roots under the three fertilization treatments compared with CK. Therefore, the function of these TFs in the delay of plant senescence would be worthy of exploration.

## 4. Materials and Methods

### 4.1. Plant Material and Fertilization Treatments

The study area was located in the Luqing Seedling Co., Ltd. Mining Village of (36.65807 N, 117.000923 E), Jinan City in Shandong Province, China. Tomato-certified variety ‘Liaofen 185’ (Liaoning Academy of Agricultural Science, Shenyang, China) was used as tomato plants. The commercial ‘Luqing’ substrate (Luqing Seedling Co., Ltd., Jinan, China) was used as the cultivation substrate. The space between rows was 110 cm and that between plants was 33 cm. The tomato plants were planted into a solar greenhouse in July 2025, and harvested in January 2026. Four experimental groups were set up as follows. The water and fertilizer were supplied uniformly among the four treatments by drip irrigation; the liquid fertilizer with the Yamazaki formula for soilless tomato culture was set as the CK. The experiment started treatment 30 days after tomato transplantation and ended 140 days after transplantation, and the details of each treatment are shown in Table 2. The T1 treatment was comprised of 7.07 mM·L^−1^ N, 2.07 mM·L^−1^ P, and 1.23 mM·L^−1^ K applied 30–60 d after planting; 3.56 mM·L^−1^ N, 1.71 mM·L^−1^ P, and 2.27 mM·L^−1^ K applied 61–80 d after planting; 13.22 mM·L^−1^ N, 2.46 mM·L^−1^ P, and 6.86 mM·L^−1^ K applied 81–120 d after planting, and 13.05 mM·L^−1^ N, 2.91 mM·L^−1^ P, and 6.47 mM·L^−1^ K applied 121–140 d after planting, which were beneficial for tomato growth according to our preliminary study. The T2 and T3 treatments were supplemented with yeast extract agents and microorganisms based on the CK, respectively (Table 2). Yeast extract was purchased from Aladdin Reagents (Shanghai, China) Co., Ltd., and used at a concentration of 20 g/L. The microorganisms used in the study were provided by Shandong Biofuture Technology Co., Ltd. (Tai’an, Shandong, China), with the main bacterial concentration ratio being: *Brevibacillus laterosporus*:*Bacillus subtilis*:*Bacillus mucilaginosus* = 7:2:1. The proportion of these biological bacteria was derived from previous research conducted in our laboratory [45]. The roots, uppermost node leaves, functional leaves, tomato fruits, and total plant samples under the four experimental groups were harvested in December 2025, respectively. Each experimental treatment has three biological replicates, with at least 50 tomato plants per replicate. 

### 4.2. Plant Trait Measurements

The dry weights of the shoots and roots were measured after being completely oven-dried at 75 °C. Root activity was expressed by TTC reduction intensity [46]. The total weight of the fruits and the number of fruits per plant were calculated at harvest time. Thirty tomato plants were selected to evaluate fruit yield/667 m^2^. 

Completely red and ripe fruits were selected for the quality parameter analysis, including the contents of vitamin C, lycopene, soluble solids, and soluble protein [46,47].

In the experiment, every 10 tomato plants were treated as one replicate, and each treatment was replicated three times.

### 4.3. RNA Extraction and Sequencing

The roots and functional leaves were frozen immediately in liquid nitrogen and stored at −80 °C for RNA extraction. The TaKaRa MiniBEST Plant RNA Extraction Kit (TaKaRa, Otsu, Shiga, Japan) was used to extract total RNA from each sample. mRNA enrichment, library construction, and high-throughput parallel sequencing were performed at the Beijing Genomics Institute (BGI, Beijing, China) with the sequencing platform BGISEQ-500 (BGI). After quality control of the raw reads and alignment and quality control of the clean reads, the differentially expressed genes (DEGs) were identified with the Q-value threshold criteria of (adjusted *p*-value) ≤ 0.05, and |log_2_ (fold change)| ≥ 1. Bioinformatics analysis was conducted as reported previously [48].

### 4.4. Validation by Quantitative Real-Time Polymerase Chain Reaction (qRT-PCR) Analysis

The PrimeScript RT Reagent Kit with the gDNA Eraser (TaKaRa) was used to reverse-transcribe 1 µg RNA into cDNA. The qRT-PCR analysis was performed using SYBR Premix Ex Taq II Kit (TaKaRa) and the LightCycler 96 system (Roche, Basel, Switzerland). The qRT-PCR primers were designed using Primer Express 3.0.1 (Table S2). The relative expression levels were calculated using the 2^−ΔΔCt^ method and were normalized using the internal reference gene *actin* (*Solyc03g078400*) [49,50].

### 4.5. Statistical Analysis

Data were expressed as mean ± standard deviation. Statistical analysis was conducted using SPSS 17.0 (SPSS, Inc., Chicago, IL, USA) software, and Duncan’s New Multiple Range test was used to compare the average differences between the different treatments. A *p*-value < 0.05 was considered significant dfference.

## 5. Conclusions

In this study, the underlying regulatory mechanisms of the three fertilization treatments to improve tomato yield and quality were explored by RNA-seq analysis. The three fertilization treatments did not change the gene expression levels in the leaves, but the signal transduction processes between the roots and leaves were significantly affected. Unlike the aboveground organs, the morphological events occurring during root senescence did not appear typical, and few studies have focused on age-related senescence in roots. According to our results, the association between root and leaf senescence is closer than we thought. Our results provide new insight into the regulation of root and leaf senescence.

## Figures and Tables

**Table 1:** KEGG pathway enrichment analysis of common upregulated and downregulated genes.

	ko04016 MAPK Signaling Pathway—Plant	ko04075 Plant-Hormone Signal Transduction
56 common down-regulated DEGs	K02183 CALM; calmodulin K13420 FLS2; LRR receptor-like serine/threonine-protein kinase FLS2 K13424 WRKY33; WRKY transcription factor 33	—
270 higher expressed genes in roots	K13413 MKK4_5; mitogen-activated protein kinase kinase 4/5 K13420 FLS2; LRR receptor-like serine/threonine-protein kinase FLS2 K13422 MYC2; transcription factor MYC2 K13424 WRKY33; WRKY transcription factor 33 K13425 WRKY22; WRKY transcription factor 22 K13447 RBOH; respiratory burst oxidase K14514 EIN3; ethylene-insensitive protein 3 K20557 VIP1; transcription factor VIP1 K20716 MAPKKK17_18; mitogen-activated protein kinase kinase kinase 17/18 K20718 ER; LRR receptor-like serine/threonine-protein kinase ERECTA	K13413 MKK4_5; mitogen-activated protein kinase kinase 4/5 K13422 MYC2; transcription factor MYC2 K14484 IAA; auxin-responsive protein IAA K14488 SAUR; SAUR family protein K14494 DELLA; DELLA protein K14514 EIN3; ethylene-insensitive protein 3
498 higher expressed genes in leaves	K02183 CALM; calmodulin K13414 MEKK1; mitogen-activated protein kinase kinase kinase 1 K13420 FLS2; LRR receptor-like serine/threonine-protein kinase K13422 MYC2; transcription factor MYC2 K13424 WRKY33; WRKY transcription factor 33 K20558 SPCH; transcription factor SPEECHLESS K20718 ER; LRR receptor-like serine/threonine-protein kinase ERECTA	K12126 PIF3; phytochrome-interacting factor 3 K13415 BRI1; protein brassinosteroid insensitive 1 K13422 MYC2; transcription factor MYC2 K14431 TGA; transcription factor TGA K14487 GH3; auxin responsive GH3 gene family K14488 SAUR; SAUR family protein K14490 AHP; histidine-containing phosphotransfer protein K14491 ARR-B; two-component response regulator ARR-B family K14494 DELLA; DELLA protein K14517 ERF2; ethylene-responsive transcription factor 2

“—” indicates that there are no relevant DEGs here.

**Table 2:** Four experimental groups set up in the study.

The Day After Planted	CK	T1 (mM·L^−1^)	T2	T3	Drip Amount (L·Plant^−1^·d^−1^)
30~60 d	Yamazaki formula (1978)	N 7.07, P 2.07, K 1.23	Yamazaki formula (1978) and yeast-extract agents	Yamazaki formula (1978) and microorganisms	0.5
61~80 d		N 3.56, P 1.71, K 2.27			0.8
81~120 d		N 13.22, P 2.46, K 6.86			1.0
121~140 d		N 13.05, P 2.91, K 6.47			0.5

## Data Availability

Original findings generated in this study are presented within the article. Additional information or clarification may be obtained by contacting the corresponding author.

## Supplementary Materials

The following supporting information can be downloaded at: https://www.mdpi.com/article/10.3390/plants15192990/s1, Figure S1: GO analysis of common DEGs with same expression trend. Figure S2: KEGG analysis of common DEGs with same expression trend. Table S1. Overview of the RNA-sequencing reads generated from each sample. Table S2. Primers for qRT-PCR validation of differentially expressed genes (DEGs).
