# Supplementary material for: Transcriptional Analysis Reveals Different Fertilizer Treatments Improve the Yield and Quality of Tomato Fruits by Postponing Age-Triggered Senescence
Source: Plants (Basel). 2026 Sep 30;15(19):2990. doi: 10.3390/plants15192990 (PMC13667078; doi:10.3390/plants15192990)
Supplement: Supplementary file 1 [file plants-15-02990-s001.zip › plants-4567827-supplementary.pdf]

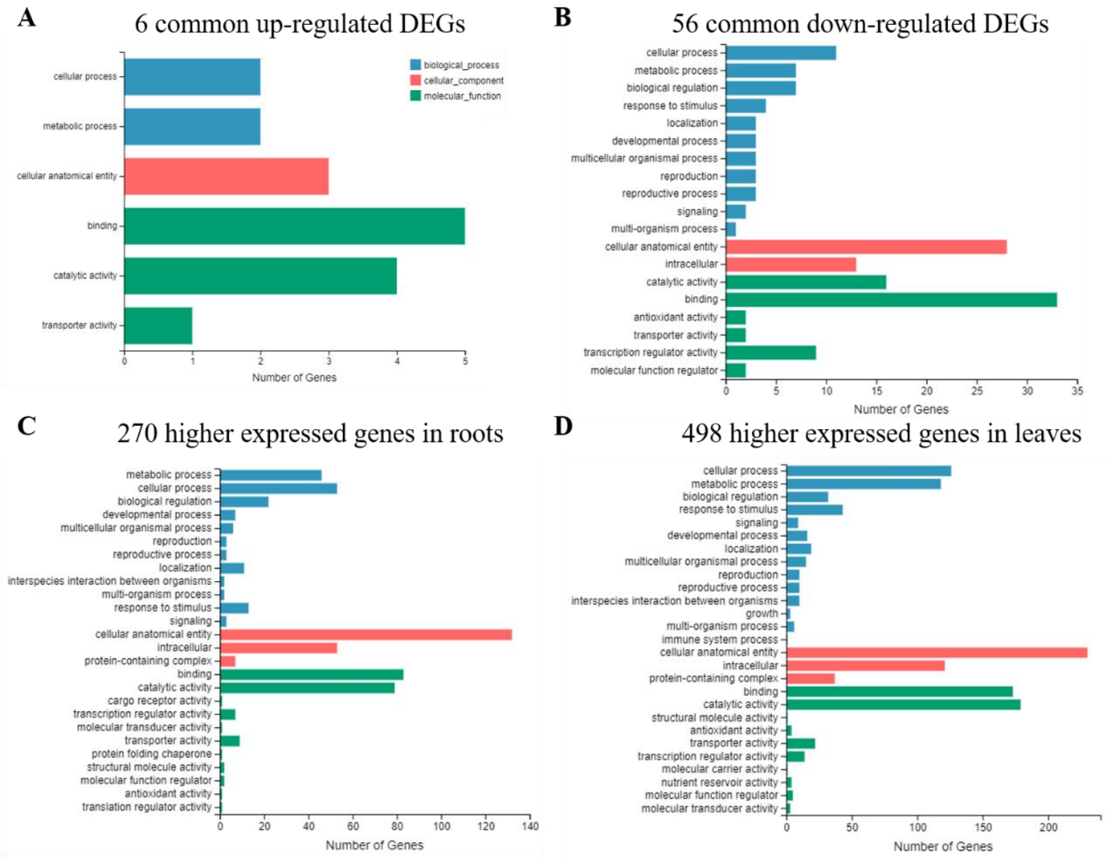

Figure S1. GO analysis of common DEGs with same expression trend.

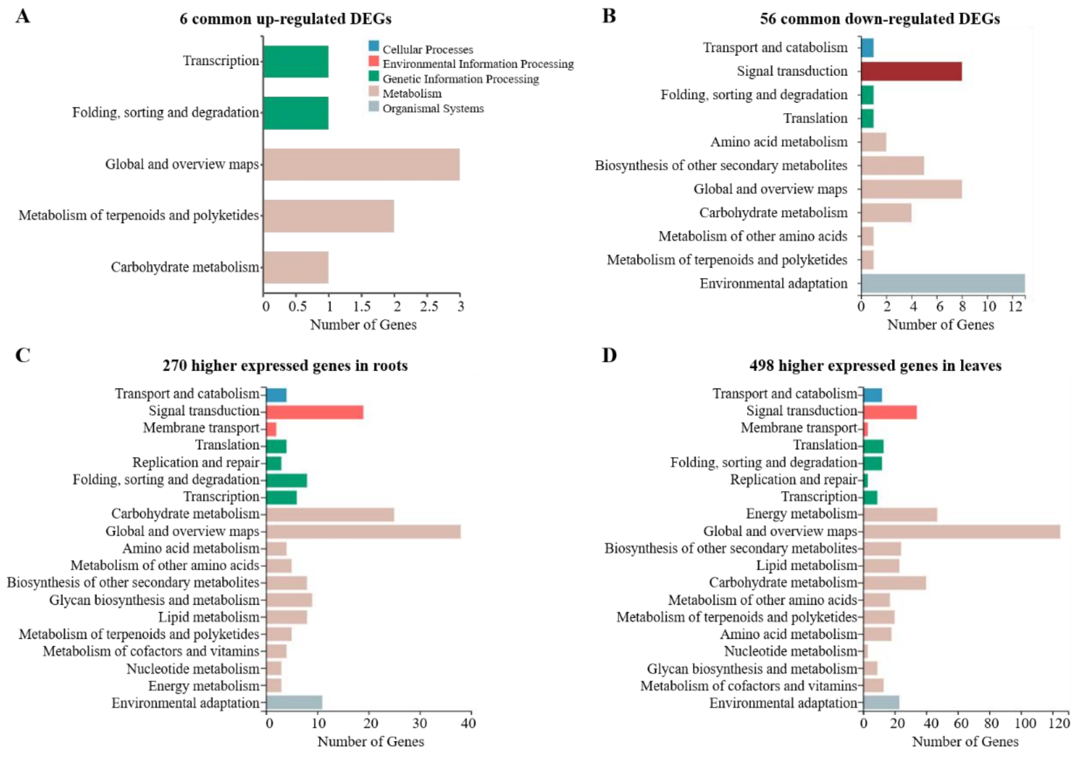

Figure S2. KEGG analysis of common DEGs with same expression trend.

Table S1. Overview of the RNA-sequencing reads generated from each sample.

Sample	Total Raw Reads (M)	Total Clean Reads (M)	Total Clean Bases(Gb)	Clean Reads Q20(%)	Clean Reads Q30(%)	Clean Reads Ratio(%)	Uniquely Mapping to genome(%)	Uniquely Mapping to gene(%)
CK-L1	23.92	23.35	1.17	97.88	92.12	97.59	86.39	78.24
CK-L2	23.92	22.72	1.14	97.9	90.32	94.96	84.57	77.22
CK-L3	21.16	20.16	1.01	97.93	90.42	95.29	85.89	78.29
CK-R1	23.92	22.94	1.15	97.62	89.45	95.88	79.37	69.25
CK-R2	23.92	22.94	1.15	97.68	89.63	95.9	78.66	68.66
CK-R3	23.92	22.9	1.14	97.5	89.04	95.72	81.89	71.99
T1-L1	23.92	23	1.15	97.58	89.34	96.12	86.91	78.64
T1-L2	23.92	22.94	1.15	97.61	89.41	95.91	86.01	78.03
T1-L3	23.92	22.89	1.14	97.53	89.15	95.7	85.76	78.6
T1-R1	21.75	21.61	1.08	97.98	93.92	99.38	85.94	73.6
T1-R2	21.75	21.6	1.08	98.11	94.32	99.3	85.6	73.16
T1-R3	21.75	21.61	1.08	98.02	94.03	99.35	84.48	72.66
T2-L1	23.92	22.69	1.13	97.87	90.25	94.86	86.24	78.84
T2-L2	23.16	22.04	1.1	97.61	89.33	95.16	85.23	77.34
T2-L3	23.92	22.78	1.14	97.73	89.85	95.21	86.44	78.65
T2-R1	23.92	22.72	1.14	97.58	89.31	94.97	80.92	70.63
T2-R2	21.75	20.99	1.05	97.52	89.15	96.52	83.36	72.84
T2-R3	23.92	22.96	1.15	97.7	89.7	95.96	81.16	71.05
T3-L1	23.92	22.77	1.14	97.84	90.1	95.17	85.43	77.72
T3-L2	23.92	22.95	1.15	97.52	89.13	95.93	85.23	78.62
T3-L3	23.92	22.92	1.15	97.57	89.28	95.79	86.05	79.2
T3-R1	23.92	22.86	1.14	97.56	89.29	95.54	83.62	73.62
T3-R2	21.75	21.65	1.08	98.32	94.84	99.56	88.06	76.17

T3-R3	21.75	21.61	1.08	98.26	94.74	99.38	84.41	72.82

Table S2. Primers for qRT-PCR validation of differentially expressed genes (DEGs)

Name	Forward primer(5'-3')	Reverse primer(5'-3')
Solyc02g062690.3 (*bHLH*)	AGACAAAGAGCAGTGTTGGAACGT	CGCAGCCGGTAATCAAATGA
Solyc03g007380.2 (*WRKY*),	ACCACGTTCTAATTCCCCTGCTAT	CGTGTTGCATGATGCTCTTTAGG
Solyc04g072070.3 (*WRKY*),	CAAATGGAGGAAATATGGGAAGAAG	TTGCAATCTCCACTTGAACACTTG
Solyc05g015840.3 (*SBP*)	CCAAGGCACCAGGGAATATAGC	GGCGATGATACTCTCTGCATTCA
Solyc06g069600.3 (*bHLH*)	TAAACGCTCGTGCAAGTGCTAAG	ACCATGGCAGGCAGAATCCT
Solyc09g014990.4 (*WRKY*)	CACAACACAGATCCAACCACCAAT	GATCCCGATGAGTTACCAAATCCT
Solyc10g008160.3 (*G2-like*)	GCACCCCTCAAATGAGAGCAT	GGAGGTTTGAGTCCTATGGGAAGTG
Solyc12g036470.2 (*bHLH*)	TGCAGAGAGCAAAGGCATATGAAG	CGGTCAATTAAAGGGCCAACTT
Solyc03g078400.3 (*Actin*)	AAGGATGCGTATGTGGGTG	GGGGTGCCTCAGTCAGGAGAACAG
